# Cyanobacterium *Nostoc* species mitigate soybean cyst nematode infection on soybean by shaping rhizosphere microbiota

**DOI:** 10.3389/fmicb.2025.1544479

**Published:** 2025-05-08

**Authors:** Chuntao Yin, Nathan Lahr, Ruanbao Zhou

**Affiliations:** ^1^North Central Agricultural Research Laboratory, US Department of Agriculture, Agricultural Research Service (USDA-ARS), Brookings, SD, United States; ^2^Department of Biology and Microbiology, South Dakota State University, Brookings, SD, United States

**Keywords:** cyanobacteria, soybean cyst nematode, nematicidal activity, microbiota, plant defense system

## Abstract

Soybean cyst nematode (SCN, *Heterodera glycines* Ichinohe) is the most devastating and yield-limiting pathogen that threatens soybean production globally. Sustainable SCN disease management strategies are needed. In this study, a cyanobacterial strain was isolated from SCN-infected soybean soil and identified as *Nostoc punctiforme* using the cyanobacterial 16S rRNA gene sequence. When susceptible soybean plants were grown in the SCN-inoculated soil, *N. punctiforme* inoculants significantly reduced the total number of SCN eggs and second-stage juveniles (J2s), compared to the control with SCN inoculation only. Further microbial analysis showed that *N. punctiforme* inoculants changed the bacterial and fungal communities in the soybean rhizospheres and significantly increased the relative abundance of several bacterial and fungal species with potential nematicidal activities, suggesting the changes of soybean rhizosphere microbiota may partially contribute to the activity of *N. punctiforme* inoculants against SCN. However, *N. punctiforme* inoculants did not directly induce soybean defense reactions against SCN. Thus, *N. punctiforme* may be a potential microbial source against SCN invasion in soybean.

## Introduction

Soybean [*Glycine max* (L.) Merr.], an economically important oilseed crop worldwide, provides protein and oil for humans and animals (SoyStats, 2020) and is considered an important contributor to meet the global food demand expected to double by 2050 (Hunter et al., 2017). However, a variety of phytopathogens cause significant yield loss and quality reduction that threaten soybean production. Soybean cyst nematode (SCN, *Heterodera glycines* Ichinohe) is the most damaging pathogen of soybean worldwide (Allen et al., 2017; Bent, 2022). SCN is widely distributed in nearly all major soybean production areas of the United States (Tylka and Marett, 2021) and causes 10–30% yield losses annually (ca. $1.5 billion) (Bandara et al., 2020; Koenning and Wrather, 2010; Tylka and Marett, 2021; Winter et al., 2006). Due to the longevity of SCN cysts in soil and unnoticeable aboveground symptoms, it is challenging to eliminate SCN from the field completely once a soybean field is infected (Arjoune et al., 2022). Genetic resistance and rotation to nonhost crops are considered primary management strategies. However, repeated monoculture of the same genetic resistance, introgressed from plant introduction (PI) 88788 and PI 548402 (Peking) (Tylka et al., 2022), has led to the evolution of SCN that overcomes the resistance (Bent, 2022). Nematicides are the other control options for soybean producers, but the negative environmental effects (Desaeger et al., 2020) and the emergence of nematicide resistance (Wram et al., 2022) have encouraged the development of eco-friendly alternatives.

Cyanobacteria, widely distributed in both aquatic and terrestrial environments, are known for their beneficial roles in agricultural ecosystems (Lee and Ryu, 2021; Parmar et al., 2023; Sithole et al., 2023). Cyanobacteria have been used for stimulating plant growth (Alvarez et al., 2024; Suresh et al., 2019) and/or for managing plant diseases (Kim, 2006; Righini et al., 2022). For example, root-drench application of cyanobacteria, *Anabaena variabilis, A. torulosa*, and *Calothrix* sp., reduced damping-off symptoms caused by soilborne pathogens like *Fusarium oxysporum, Pythium debaryanum*, and *Rhizoctonia solani* in tomato and cotton (Chaudhary et al., 2012; Prasanna et al., 2013; Righini et al., 2022). Recently, the use of cyanobacteria-based biostimulants to combat plant-parasitic nematodes has gained attention (Sithole et al., 2023). *Synechococcus nidulans* increased nematode J2 mortality and inhibited egg hatching of multiple nematodes, including *Meloidogyne graminicola, M. incognita, Heterodera cajani, H. avenae*, and *Rotylenchulus reniformis* (Holajjer et al., 2012). *Anabaena oryzae* had nematicidal activity against *Meloidogyne incognita* on banana (*Musa acuminata*) plants (Hamouda and El-Ansary, 2017). The aqueous and methanolic extracts of cyanobacteria *Oscillatoria* sp. were demonstrated to decrease the volume of galls, eggs, and adult female root-knot nematode (*M. incognita*) in soybean roots and number of J2s in soil (Ghareeb et al., 2022). Studies on the suppression of cyanobacteria on plant pathogenic nematodes are still in their infancy, despite very promising results being reported.

Some cyanobacterial species can protect host plants against pathogenic nematodes via nematocidal activity (Choleva et al., 2007; Ghareeb et al., 2022; Hamouda and El-Ansary, 2017). Holajjer et al. (2013) noted that cyanobacteria produce cyanotoxins to alter nematode behavior or disrupt egg hatching process. Previous studies have revealed that the phytoactive metabolites of cyanobacteria, such as amides, alkaloids, saponins, and carotenoids, slowed the life cycle of *Caenorhabditis elegans* (Asimakis et al., 2022; Biondi et al., 2004). Ghareeb et al. (2022) found that *Oscillatoria* sp. activated soybean defense system against invasion of *M. incognita*. However, the detailed mode of action of cyanobacteria on root pathogens is largely unexplored.

Several studies have shown that cyanobacterial inoculants influenced soil- and plant-associated microbes. Cyanobacterium (*Calothrix elenkenii*) increased the population densities of culturable microbiome members from plant tissues, with about 60% culturable bacterial isolates belong to *Bacillaceae* (Priya et al., 2015). Foliar and soil drench of *Bacillus* sp. and *Nostoc-Anabaena* consortium altered *nifH* and bacterial *amoA* gene abundances (Thapa et al., 2021). Cyanobacteria-green microalgae consortia inoculants shifted bacterial and fungal communities of chili plants, with some microbial taxa (Firmicutes, Chloroflexi, Planctomycetes, Proteobacteria, Bacillariophyta, Basidiomycota, and Glomeromycotan) dominating in the consortia-treated soils, while others (Actinobacteria, Bacteroidetes, and Streptomycota) dominating in untreated soils (Jose et al., 2024). Surprisingly, little information is available on cyanobacteria-mediated microbiota against soilborne pathogens.

In the present study, we isolated a cyanobacterium from SCN-infected soybean soil and identified it to be *Nostoc punctiforme* using the cyanobacterial 16S rRNA gene-specific primers. The objectives of this study were: 1. to evaluate the effect of the isolated *N. punctiforme* on SCN infestation; 2. to examine the effect of *N. punctiforme* inoculants on soybean rhizosphere microbiota; and 3. to assess the ability of *N. punctiforme* to induce soybean systemic resistance against SCN. We hypothesized that *N. punctiforme* inoculants would mitigate SCN damage to soybean by changing soybean rhizosphere microbiota and inducing soybean systemic resistance.

## Materials and methods

### Plant, soil, and soybean cyst nematode

Soybean cultivar “Williams 82,” susceptible to SCN, was used in this study. Soil was collected from the field plots with a maize-soybean rotation since 2018 at the Eastern South Dakota Soil and Water Research Farm (44°19′N, 96°46′W) in Brookings, SD. Soil was collected at a depth of 0 to 20 cm using a shovel in the fall of 2021 after crop harvest and before ground freezing and transferred to the lab. The soil was mixed thoroughly and sieved through a 5 mm aperture sieve to remove plant debris and rocks, and then stored at 4°C for further use. The collected field soil was mixed with sterile quartz sand (4030 silica sand, 0.45–0.55 mm diameter, Unimin Minnesota Corp, Le Sueur, MN) and calcined clay (Turface All Sport Pro, Profile Products, Buffalo Grove, IL) in a ratio of 1:1:1 (w/w/w) for SCN population increase. Soybean seeds were sterilized by exposure to chlorine gas [25:1 (v/v) 10% sodium hypochlorite and 12 N HCl] for 16 h (Chen et al., 2018), planted in the mixed soil, and grown in a growth chamber with a 16 h photoperiod (light intensity 1,000 μE m^−2^ s^−1^) at 26 ± 2°C. SCN HG type 7 was initially isolated from the soybean field in Brookings, SD, and multiplied on “Williams 82” in a growth chamber as previously described (Yin et al., 2024). SCN Cysts and eggs were collected from the soybean roots and soil according to the technique of Shepherd (1970) with modifications as previously described (Yin et al., 2024).

### Cyanobacterial strain isolation and culture conditions

Among the planting pots for SCN population increase, one pot with lower SCN populations displayed blue-green color in the soil that appears to be cyanobacterial-like microbe under a compound microscope (Leica Microsystems Inc., Deerfield, IL). To isolate the blue-green organism, 100 μl of SCN egg extracts were spread over the surface of BG11 medium, a cyanobacterial selection medium (Ferris and Hirsch, 1991), in petri dishes, and then incubated in a growth chamber with a 16 h photoperiod at 26 ± 2°C. After the growth of the blue-green cultures, a series of successive streaking (5–6 times) of the blue-green cultures was performed in BG11 medium and the purity of the culture was ensured by regular observation under a compound microscope (Wang et al., 2018). The blue-green organism was then re-streaked in BG11_0_ medium (no combined nitrogen in BG11 medium) for 3–4 times. The blue-green strain was able to reproducibly grow well in BG11_0_ medium, suggesting that it has nitrogen-fixing ability.

### Genomic DNA extraction and cyanobacterium identification

Genomic DNA (gDNA) of the isolated culture was extracted following the protocol of cyanobacterial gDNA extraction as previously described (Qiu et al., 2019). Briefly, 200 ml of cultures (OD_720_ = 0.4) were centrifuged and 500 μl of the sterilized 10% sucrose buffer (50 mM Tris-HCl, pH 8.0, 10 mM EDTA), 50 μl of 125 mg ml^−1^ lysozyme (MilliporeSigma, Rockville, MD), 150 μl of 10% (w/w) SDS, and 10 μl of RNase (10 mg ml^−1^) were added to suspend/wash the cell pellets. The suspension was incubated at 37°C for 1 h. Then the saturated phenol and chloroform solution was used to extract gDNA. The gDNA was dissolved in sterile ddH_2_O for further use.

The 16S rRNA gene fragment was amplified from the extracted gDNA using cyanobacterial-specific primers (CYA106F 5′-CGGACGGGTGAGTAACGCGTGA-3′ and CYA781R 5′-GACTACWGGGGTATCTAATCCCWTT-3′) as previously described (Boutte et al., 2006; Nübel et al., 1997). PCR reaction consisted of 1 μl of gDNA (50 ng), 10 μl of 2x Phusion master mix (ThermoFisher Scientific Inc., MA), and 0.25 μM of each primer in a total volume of 20 μl. PCR was performed as follows: 98°C for 30 s; 30 cycles of 98°C for 10 s, 55°C for 10 s, and 72°C for 45 s; final extension at 72°C for 10 min. The PCR products were analyzed on a 1.5% agarose gel, purified with the GeneJET PCR purification kit (ThermoFisher Scientific Inc., MA), and sequenced from both ends at Elim Biopharm (Hayward, CA). The sequences were subjected to a BLAST analysis at NCBI database and the assembled sequence was deposited in NCBI database (accession number: PQ740210).

### SCN inhibition assay

The efficacy of the isolated cyanobacterium against SCN HG type 7 in soybean “Williams 82” was examined in a growth chamber. The collected field soil was mixed with sterile quartz sand in a ratio of 2:3 [w(fresh weight)/w(dry weight)]. Plastic cones (40 mm in diameter and 210 mm long) were filled with 120 g of mixed soil. One pre-germinated “Williams 82” seed was sown in each cone, followed by a 15 ml aliquot of the isolated cyanobacterial culture suspended in sterile ddH_2_O. Cyanobacterial culture was added with high concentration (OD_720_ = 0.5) or low concentration (5x dilution, OD_720_ = 0.1). Finally, 4,000 SCN eggs (Haarith et al., 2021) were added in a 3 cm deep hole around the soybean plant. Sterile ddH_2_O or sterile ddH_2_O and SCN were used as controls. Each treatment had four replicates. Cones were arranged in a randomized complete block design in plastic racks and maintained in the controlled conditions (16 h photoperiod, 26 ± 2°C). Each cone received 10 ml of water daily in the first 3 weeks, then 10 ml of water twice a day in the final 2 weeks. After 35 days, the soybean plants were uprooted gently and washed free of adhering soil. Plant parameters, including shoot length and fresh shoot weight, were measured. The SCN eggs and J2s were extracted from the soil and half of plant roots (Shepherd, 1970), and the number of eggs and J2s was counted using a Leica DML compound microscope at 100 x magnification (Leica Microsystems Inc., Deerfield, IL). The other half of the roots were used for rhizosphere soil preparation as described previously (Yin et al., 2021). The roots after washing off rhizosphere soil were stored at −80°C for RNA extraction. The experiment was repeated three times and the rhizosphere DNA and root RNA were prepared from one experiment with four replicates.

### Soil DNA extraction and amplicon sequencing of rhizosphere microbiota

Amplicon sequencing was used to evaluate whether the cyanobacterial inoculants affect the rhizosphere microbiota in soybean plants grown in the SCN-inoculated soil. Rhizosphere soil DNA was extracted using a DNeasy PowerSoil kit (Qiagen, Carlsbad, CA) and a bead beater SPEX 1600 MiniG (Spex SamplePrep, Metuchen, NJ) at 1,500 Hz for 1 min. The DNA was quantified using a Nanodrop spectrophotometer (Thermo Fisher Scientific, Waltham, MA) and sent to the University of Minnesota Genomics Center for amplification and sequencing. For bacterial amplicon sequencing, v4 hypervariable region of the 16S rRNA gene was amplified using forward primer 515f (GTGYCAGCMGCCGCGGTAA) and reverse primer 806r (GGACTACNVGGGTWTCTAAT) (Caporaso et al., 2011). For fungal amplicon sequencing, fungal internal transcribed spacer 1 (ITS1) region was amplified using a two-step dual-indexed amplification (Gohl et al., 2016a,b). The fungal ITS1 region was amplified in the first set of PCR using primers ITS1*_Nextera (5′-TCGTCGGCAGCGTCAGATGTGTATAAGAGACAGCTTGGT CATTTAGAGGAAG*TAA-3′) and ITS2 (5′-GTCTCGTG GGCTCGGAGATGTGTATAAGAGACAGGCTGCGTTCTTCAT CGA*TGC-3′) (ITS-specific sequences in bold, “^*^” indicates the position of a phosphorothioate bond) (Gohl et al., 2016b). PCR assays were carried out using KAPA HiFi PCR kit (Roche, Indianapolis, IN, USA) and an initial denaturing at 95°C for 5 min, followed by 25 cycles of denaturation at 98°C for 20 s, annealing at 55°C for 15 s, and extension at 72°C for 1 min, with a final extension at 72°C for 5 min. PCR products were diluted to 1:100, and 5 μl of diluted PCR product was included in a second round of PCR using forward indexing primer (5′-AATGATACGGCGACCACCGAGATCTACACTCGTCGGC AGCGTC-3′) and reverse indexing primer (5′-CAAGCAGAAGACGGCATACGAGATGTCTCGTGGGCTCGG-3′) (Gohl et al., 2016b). The second PCR consisted of an initial denaturation at 95°C for 5 min, followed by 10 cycles of 98°C for 20 s, 55°C for 15 s, and 72°C for 1 min, with a final extension at 72°C for 5 min. Then, the amplicons were pooled, size selected, spiked with 20% PhiX, and sequenced (2 × 300 paired-end, V3 chemistry) on the Illumina MiSeq platform.

### Amplicon sequence processing

The sequence processing was conducted using USEARCH (v11) (Edgar, 2010, 2018) to denoise sequences and define zero-radius operational taxonomic units (ZOTUs, 100% identity threshold). Briefly, primer and barcode sequences were removed along with 10 and 55 bp for bacterial sequences and 30 and 85 bp for fungal sequences from forward and reverse reads, respectively. Reads were paired with 15 maximum differences and an 80% identity threshold. To generate high-quality reads for denoising, reads were filtered with a maximum expected error rate of 1, singletons were removed, and sequences were denoised using the “unoise3” algorithm (Edgar, 2016). Processed reads were then mapped to ZOTU representatives to generate a ZOTU abundance table. Taxonomy was assigned to ZOTUs using the SINTAX algorithm with an 80% confidence threshold to the Ribosomal Database Project reference database (v18, Cole et al., 2014) for bacterial sequence and the UNITE reference database (v7, Tedersoo et al., 2018) for fungal sequence. ZOTUs identified as non-bacteria or non-fungi were discarded, and ZOTU tables that were rarefied were subsampled in place of rarefied sequences for all analyses unless otherwise noted. The sequencing data was deposited in NCBI Sequence Read Archive with the project PRJNA1196987 and available after the acceptance of the manuscript.

### RNA extraction from soybean roots, cDNA synthesis, and quantitative PCR assay

To examine whether the isolated cyanobacteria inoculants induce plant defense system against SCN in soybean roots, the expression of five defense-related genes (*PR1, PR2, PR3, PR5*, and PR10) was measured by RT-quantitative PCR (qPCR) using gene-specific primers and the soybean ubiquitin gene was used as a reference (Supplementary Table 1). Total RNA was extracted from the soybean roots using RNeasy plant kit (Qiagen, Carlsbad, CA) according to manufacturer's instruction. All RNA samples were digested with DNase I (New England Biolabs, Ipswich, MA) following the manufacturer's instruction prior to synthesizing cDNA. The absence of gDNA contamination was subsequently confirmed by the lack of PCR amplification of RNA samples with primers designed for soybean ubiquitin gene. First-strand cDNA was synthesized using ProtoScript first strand cDNA synthesis kit (New England Biolabs, Ipswich, MA). qPCR was performed using SsoAdvanced Universal SYBR Green Supermix kit (Bio-Rad, Hercules, CA). qPCR reaction consisted of 10 μl PCR reaction by mixing 5 μl of SYBR Green Supermix (2x), 0.4 μl of each primer (10 μM), and 4.2 μl of diluted cDNA. qPCR was conducted on CFX Opus Real-Time PCR system (Bio-Rad, Hercules, CA) using the following conditions: for the first cycle, samples were initially incubated for 30 s at 94°C, followed by 45 cycles at 94°C for 15 s, and 60°C for 20 s, followed by a melt curve of 65–95°C with 0.5°C increments every 5 s.

### Statistical analysis and data visualization

The effect of the isolated cyanobacterium on the number of SCN eggs and J2s extracted from soil and soybean roots was evaluated using Kruskal-Wallis's test followed by Wilcoxon *post hoc* tests in JMP Pro 15.1.0 (SAS Institute, Cary, NC). Microbiome data analysis was performed in R version 4.3.2 using multiple packages, including vegan (v2.6.8, Oksanen et al., 2024), pheatmap (v 1.0.12, Kolde, 2015), and ggplot2 (v3.5.1, Wickham, 2016). Microbial alpha diversity was calculated using the “diversity” function of the vegan package. After normality and homogeneity of variance testing (Shapiro-Wilks and Levene's test), the difference in diversity index among treatments was examined with Kruskal-Wallis test, followed by a *post hoc* Dunn's test using the FSA package. Nonmetric multidimensional scaling (NMDS) was used to visualize the similarity of microbial community among rhizosphere samples based on Bray-Curtis distances. Permutational multivariate analysis of variance (PERMANOVA) was performed to determine microbial differences due to cyanobacteria and SCN inoculation. DESeq2 (v1.46.0, Love et al., 2014) was performed to identify microbial ZOTUs that differed in the rhizosphere samples between cyanobacteria-treated and -untreated samples with ZOTUs tables. Briefly, the ZOTUs tables were filtered to remove rare taxa (<10 total sequences). Those ZOTUs were kept that had normalized counts >10 and that were present in three or more samples. Wald's test was used to contrast ZOTUs from cyanobacterium-treated and -untreated samples or from high concentration of cyanobacterium and low concentration of cyanobacterium. ZOTUs were considered differentially abundant if they had a base mean >20, FDR adjusted *p* < 0.1, and estimated log2-fold change >1. Relative abundance of differential ZOTUs was then plotted in a heatmap using DESeq2 normalized log (x + 1) transformed counts.

## Results

### Identification of the isolated cyanobacterium

The isolated cyanobacterium grew well autotrophically in the nitrogen-free medium BG11_0_, which suggested it is capable of nitrogen fixation. Under microscopic observation, the isolated cyanobacterium is a filamentous organism (Supplementary Figure 1) and can produce heterocyst cells (Supplementary Figure 1B). The 16S rRNA gene was amplified from the extracted gDNA of the cyanobacterial cultures using cyanobacterial-specific primers and sequenced. The 16S rRNA gene fragment was 641 bp in length and was deposited in GenBank (accession number: PQ740210). BLAST results showed that the sequence of the isolated cyanobacterium was 99.68% identical to *Nostoc punctiforme* (Sequence ID: MH090930.1).

### Effect of *Nostoc punctiforme* on SCN population and soybean growth

**Figure 1:**
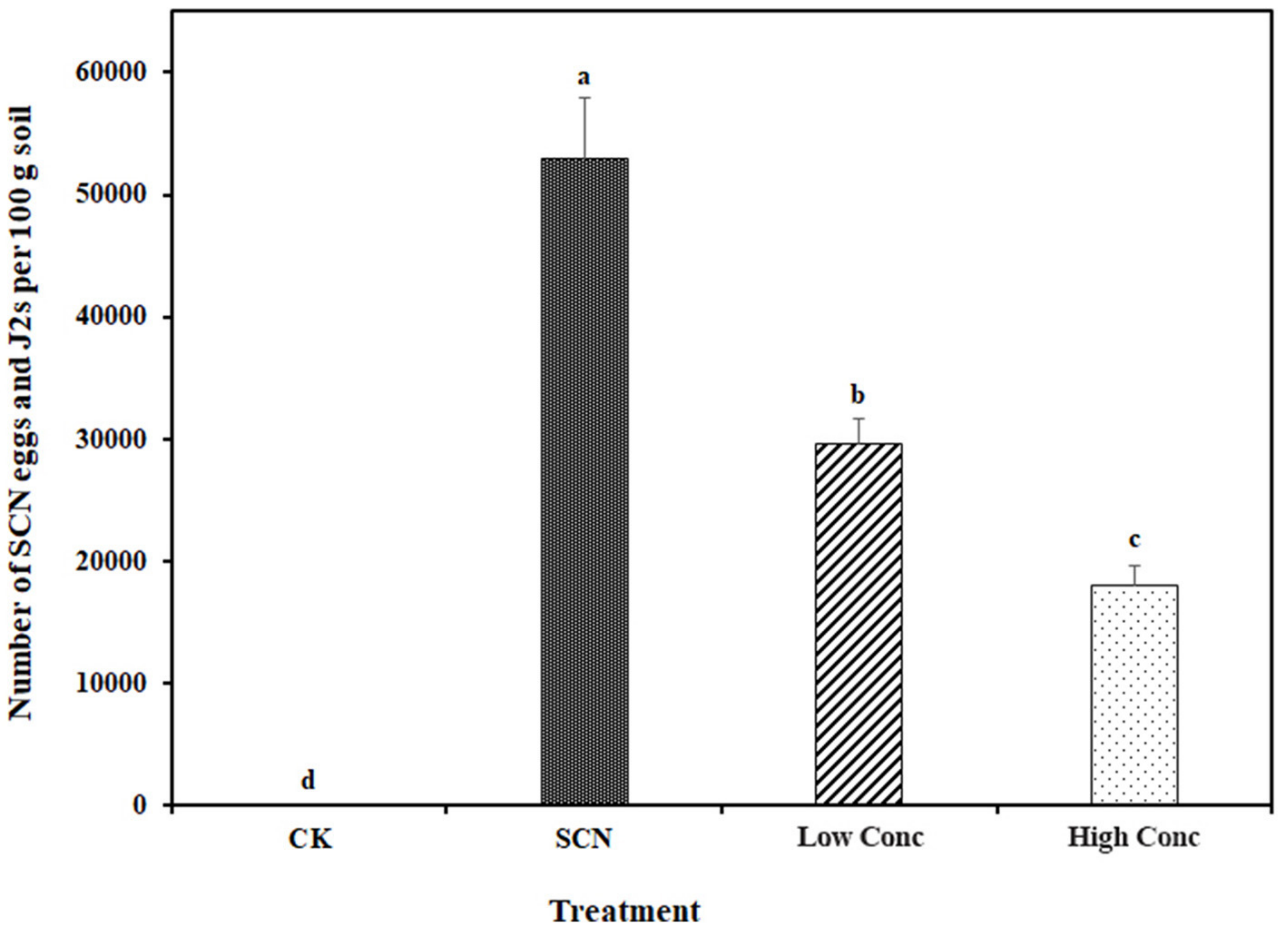
Effect of *Nostoc punctiforme* inoculants on soybean cyst nematode. CK: control without SCN (HG type 7) and *N. punctiforme* inoculation, SCN: SCN inoculation, Low Conc: SCN and low concertation *N. punctiforme* inoculation, High Conc: SCN and high concertation *N. punctiforme* inoculation. Different letters indicate a statistically significant difference between treatments within an experiment as determined by Kruskal-Wallis's test (*p* ≤ 0.05, *n* = 4).

After soybean plants grew in the SCN and *N. punctiforme* inoculated soil for 35 days*, N. punctiforme* inoculants significantly reduced the total number of SCN eggs and J2s, compared to SCN inoculation only. But the number of SCN eggs and J2s was still significantly higher than the control without SCN infestation (Figure 1 and Supplementary Figure 2). Additionally, the inoculation concentration of *N. punctiforme* appeared to affect the SCN suppression (Figure 1). However, we did not observe that *N. punctiforme* inoculants significantly improved soybean plant growth in the experimental conditions, with plant shoot length and fresh weight similar to that of the SCN infestation (Supplementary Figure 3).

### Effect of *Nostoc punctiforme* on soybean rhizosphere microbiota

Bacterial and fungal communities from the soybean roots across all samples were characterized. Bacterial communities in the soybean rhizosphere were dominated by phyla Proteobacteria (35.53%), Actinobacteria (26.59%), and Acidobacteria (10.97%) (Supplementary Figure 4A). The most abundant fungal phyla in the soybean rhizosphere were Ascomycota (50.38%), followed by Basidiomycota (38.45%), Chytridiomycota (5.18%), and Mortierellomycota (4.22%) (Supplementary Figure 4B).

The *N. punctiforme* inoculants did not influence the alpha diversity indexes of bacterial community in the soybean rhizosphere, including Shannon, richness, and inverse Simpson, except for the low concentration of *N. punctiforme* inoculants effects on inverse Simpson. In contrast, the *N. punctiforme* inoculants significantly decreased the Shannon diversity of fungal community, regardless of the inoculation concentration, and decreased the richness of fungal community in the low concentration inoculation (Supplementary Table 2) with no effects on inverse Simpson indexes.

**Figure 2:**
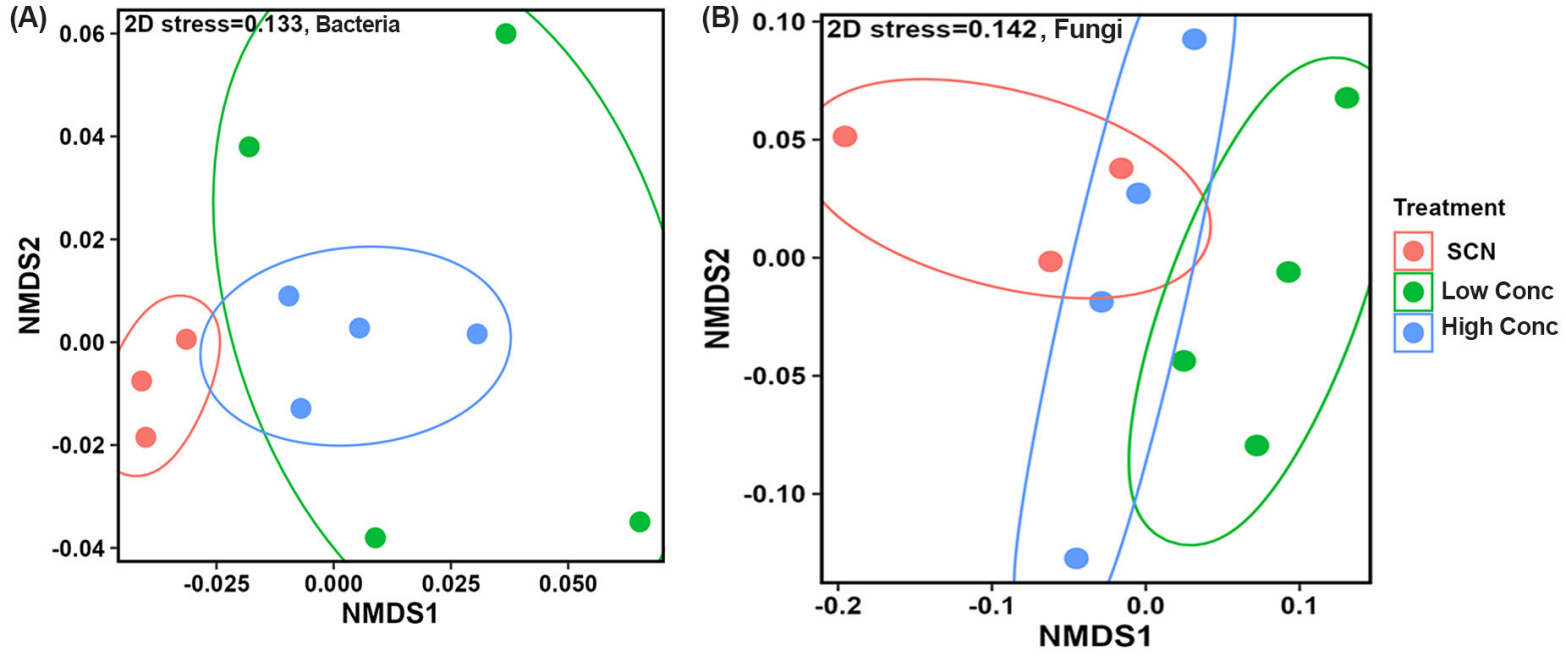
Nonmetric multidimensional scaling (NMDS) plots of Bray-Curtis distances among soybean rhizosphere microbiota following SCN and *N. punctiforme* inoculation. Samples are colored by SCN and *N. punctiforme* inoculation. **(A)** bacterial community, **(B)**. fungal community. SCN: SCN inoculation, Low Conc: SCN and low concertation *N. punctiforme* inoculation, High Conc: SCN and high concertation *N. punctiforme* inoculation. The different groups are highlighted by ellipses showing a 95% confidence range for the variation within each group.

Non-metric multidimensional scaling (NMDS) analysis revealed that the *N. punctiforme* inoculants changed the bacterial and fungal communities in the soybean rhizosphere (Figures 2A, B). Bacterial community clustered by *N. punctiforme* inoculation treatments, regardless of inoculation concentration. *N. punctiforme* inoculants and their concentration separated fungal community. Further permutational multivariate analysis of variance (PERMANOVA) supported the effect of the *N. punctiforme* inoculants on soybean rhizosphere microbiota (Table 1). The *N. punctiforme* inoculants had significant effects on the bacterial (*p* = 0.005) and fungal communities (*p* = 0.017), explaining 24.9% of the overall variation in the bacterial community and 35.3% in the fungal community, respectively (Table 1).

**Table 1:** PERMANOVA of impacts of cyanobacteria inoculation on soybean rhizosphere microbiota.

**Microbiome**	**Factor**	***F* value**	***r* ^2^**	***p* value^a^**
Bacterial community	Cyanobacteria	1.490	0.249	**0.005**
Fungal community	Cyanobacteria	2.184	0.353	**0.017**

a Values in bold represent factors with a significant effect (*p* < 0.05).

**Figure 3:**
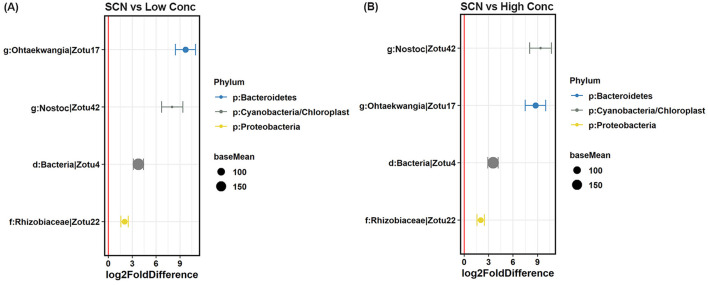
Differentially abundant bacterial ZOTUs identified in the soybean rhizosphere by SCN and *N. punctiforme* inoculations (FDR adjusted *p* < 0.1, Wald's test). **(A)** Bacterial ZOTUs in the SCN and low concertation *N. punctiforme* inoculation, **(B)** bacterial ZOTUs in the SCN and high concertation *N. punctiforme* inoculation. SCN: SCN inoculation, Low Conc: SCN and low concertation *N. punctiforme* inoculation, High Conc: SCN and high concertation *N. punctiforme* inoculation. Values on the x-axis presented the DESeq2-estimated log2-fold difference. The red vertical line represents a zero-fold change, where ZOTUs to the right of the line (positive values) are increased in relative abundance in *N. punctiforme* inoculation, and those to the left of the line (negative values) are more abundant in no *N. punctiforme* inoculation. Dots indicate ZOTUs, where the size of the dot is scaled by its mean abundance among all samples (base mean ≥ 20) and its color represents the phylum to which that ZOTUs belongs.

**Figure 4:**
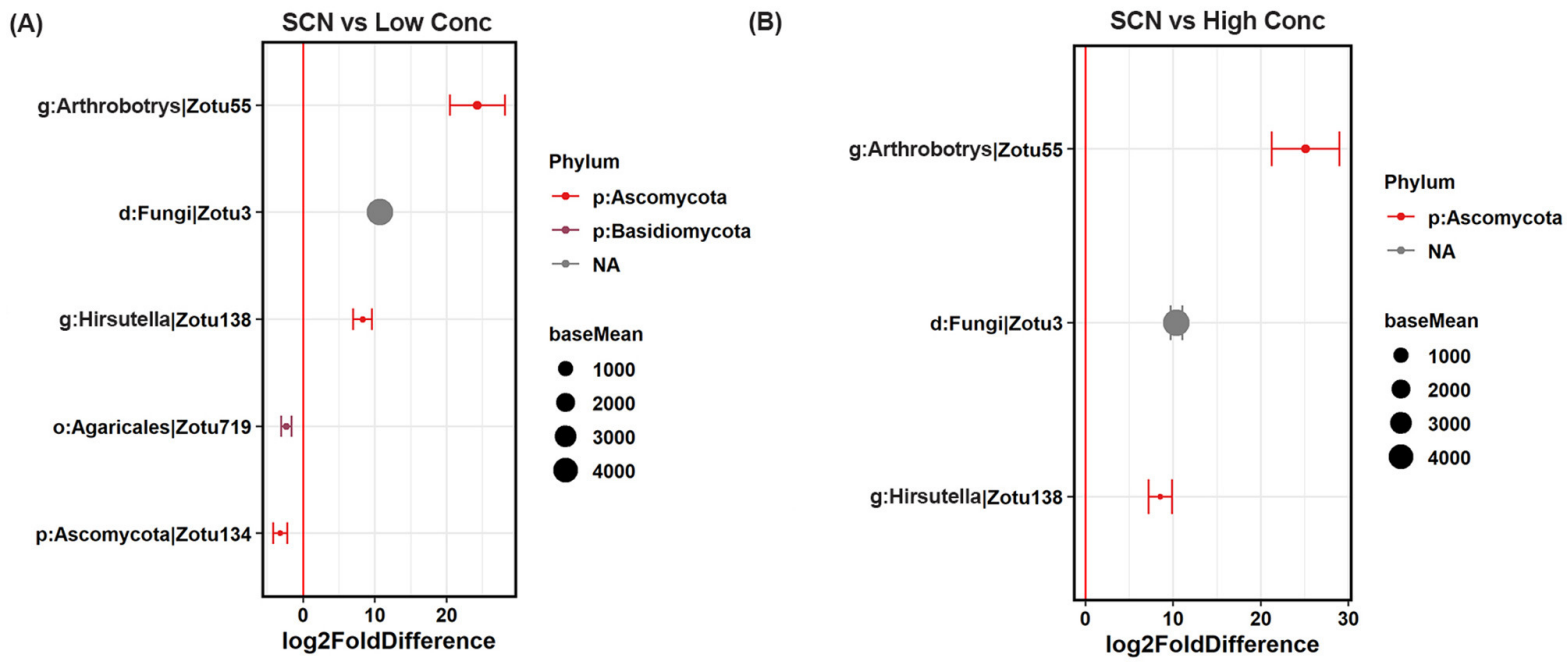
Differentially abundant fungal ZOTUs identified in the soybean rhizosphere by SCN and *N. punctiforme* inoculations (FDR adjusted *p* < 0.1, Wald's test). **(A)** Fungal ZOTUs in the SCN and low concertation *N. punctiforme* inoculation, **(B)** fungal ZOTUs in the SCN and high concertation *N. punctiforme* inoculation. SCN: SCN inoculation, Low Conc: SCN and low concertation *N. punctiforme* inoculation, High Conc: SCN and high concertation *N. punctiforme* inoculation. Values on the x-axis presented the DESeq2-estimated log2-fold difference. The red vertical line represents a zero-fold change, where ZOTUs to the right of the line (positive values) are increased in relative abundance in *N. punctiforme* inoculation, and those to the left of the line (negative values) are more abundant in no *N. punctiforme* inoculation. Dots indicate ZOTUs, where the size of the dot is scaled by its mean abundance among all samples (base mean ≥ 20) and its color represents the phylum to which that ZOTUs belongs.

Most interestingly, the *N. punctiforme* inoculants significantly increased the relative abundance of four bacterial species (ZOTUs), including ZOTU17 belonging to genus *Ohtaekwangia*, ZOTU22 (family Rhizobiaceae), ZOTU42 (genus *Nostoc*), and unidentified bacterial species ZOTU4, compared to SCN inoculation only, regardless of the *N. punctiforme* inoculation concentration (Figure 3). Furthermore, the relative abundance of three fungal species was higher in the *N. punctiforme* inoculated soybean rhizosphere than that without *N. punctiforme* inoculants, and they were ZOTU55 classified as genus *Arthrobotrys* (family Orbiliaceae), ZOTU138 belonging to genus *Hirsutella* (family Ophiocordycipitaceae), and unidentified fungal species ZOTU3, regardless of the *N. punctiforme* inoculation concentration (Figure 4).

### Effect of *N. punctiforme* inoculants on expression of soybean defense-related genes

**Figure 5:**
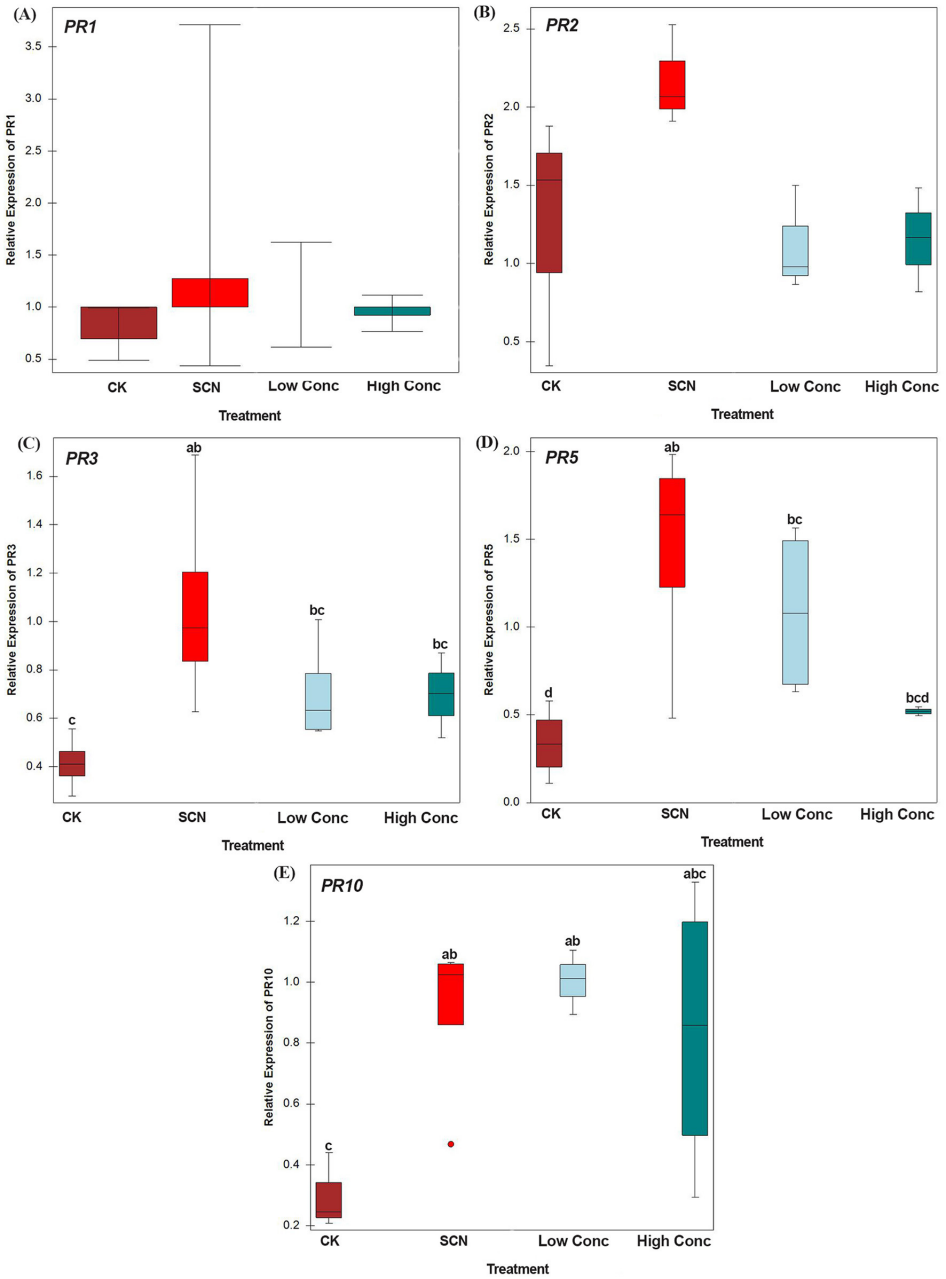
RT-qPCR analysis of the expression of pathogenesis-related genes. **(A)** Relative expression of *PR1*, **(B)** Relative expression of *PR2*, **(C)** Relative expression of *PR3*, **(D)** Relative expression of *PR5*, **(E)** Relative expression of *PR10*. Gene expression levels were analyzed from four treatments: CK: control without SCN and *N. punctiforme* inoculations, SCN: SCN inoculation, Low Conc: SCN and low concertation *N. punctiforme* inoculations, High Conc: SCN and high concertation *N. punctiforme* inoculations. Data are normalized by the ubiquitin reference gene and relative transcript levels in comparison with those of CK. Error bars represent standard error of the mean values of two technical replicates from four biological replicates. Differences in expression levels were tested by analysis of variance (ANOVA) and Tukey test. For each gene, bars with different letters indicate statistically significant differences between treatments.

SCN inoculation significantly increased the expression of three soybean defense-related genes, including *PR3, PR5*, and *PR10*, but did not affect the expression of *PR1* and *PR2*, compared to no SCN inoculation. However, we did not observe that *N. punctiforme* inoculants significantly impact the expression of all five tested *PR* genes, compared to SCN inoculation only (Figure 5).

## Discussion

In this study, a cyanobacterium was isolated from the SCN-infected soybean soil and further identified as *Nostoc punctiforme* using cyanobacterial-specific 16S rRNA gene sequence. The *N. punctiforme* inoculants significantly reduced SCN population when soybean “Williams 82” was grown in the SCN-infested soil, compared to the control (no *N. punctiforme* additions). The results suggested that the isolated *N. punctiforme* may have antagonistic activity and could be considered as a potential microbial candidate for enhancing soybean plants against SCN. Prokaryotic cyanobacteria possessing beneficial traits, such as fixing atmospheric nitrogen, promoting plant growth, and increasing soil pores by producing adhesive substances (Kuraganti et al., 2020), are abundant in agricultural soils. Some cyanobacteria have been utilized as biofertilizers to enhance soil fertility and to promote crop growth in cropping systems (Joshi et al., 2020; Ramakrishnan et al., 2023). However, adding the isolated *N. punctiforme* cultures into the soil did not significantly increase soybean seedling biomass or promote the growth of wheat and maize plants compared to no *N. punctiforme* inoculants (data not shown).

The insecticidal properties of several cyanobacteria and their extracts have been used as environmentally friendly approaches in integrated pest management for pest control (Asimakis et al., 2022; Ghareeb et al., 2022). Although numerous studies demonstrated that inoculating soil-friendly microorganisms, like biocontrol microbial strains (*Bacillus* spp. and *Trichoderma* spp.), altered microbial diversities and activities (Li et al., 2022; Saravanakumar et al., 2017; Senkovs et al., 2021), there was little information about cyanobacterial inoculant effects on microbiomes. In this study, we found that *N. punctiforme* inoculants significantly shifted rhizosphere microbial communities in soybean grown in the SCN inoculated soil. Notably, *N. punctiforme* inoculants significantly increased the relative abundance of four bacterial species (ZOTU4, ZOTU17, ZOTU22, and ZOTU42) and three fungal species (ZOTU55, ZOTU138, and ZOTU3), regardless of the inoculation concentration. High abundance of ZOTU42, which belongs to genus *Nostoc*, in the *N. punctiforme* inoculated samples was expected. ZOTU17 and ZOTU22 belong to bacterial genus *Ohtaekwangia* (family Cytophagaceae) and family Rhizobiaceae, respectively. The two bacterial species are involved in soil nitrogen cycling (Alves et al., 2014; Rodríguez-Caballero et al., 2017). It is interesting to note that *Ohtaekwangia* can produce marinoquinoline, a chemical compound with antibiotic, antifungal, and insecticidal activities that could protect plants from pathogens and predators (Okanya et al., 2011). Further, Deng et al. (2021) found that *Ohtaekwangia* was the dominant bacterial genus in the suppressive soil against pathogenic fungal *Fusarium oxysporum* and Ou et al. (2019) showed that OTU belonging to genus *Ohtaekwangia* was negatively correlated with the relative abundance of *F. oxysporum*, demonstrating *Ohtaekwangia* being involved in fungal disease suppression. ZOTU55 and ZOTU138 belong to fungal genus *Arthrobotrys* (family Orbiliaceae) and *Hirsutella* (family Ophiocordycipitaceae), respectively. *Arthrobotrys* spp. are a well-known nematode-trapping fungus with biocontrol potential against root-knot nematodes (Eken et al., 2023; Yu et al., 2014). *Arthrobotrys* spp. can immobilize nematodes remotely through secreting specific nematotoxic metabolites (Nordbring-Hertz, 2004). *Arthrobotrys* spp. widely distribute in most habitats due to their adaptability and flexible lifestyles, thus they are considered excellent agents for controlling plant parasitic nematodes (Eken et al., 2023; Soliman et al., 2021). But there is no report that *Arthrobotrys* spp. can inhibit soybean cyst nematodes. *Hirsutella* spp. are another most discussed fungi for their biological control of nematodes, including SCN (Chen and Liu, 2007; Haarith et al., 2021; Sun et al., 2024). Among them, two species, *H. rhossiliensis* and *H. minnesotensis*, have been extensively studied on their SCN control abilities (Chen and Liu, 2007). *H. minnesotensis* was detected in 14% of soybean fields in Minnesota, South Dakota, and Michigan in the US (Chen and Liu, 2007; Mwaheb et al., 2017) and parasitizes SCN juveniles and other vermiform motile stages using adhesive conidia that penetrate and eventually kill the nematodes (Chen and Liu, 2007). ZOTU4 and ZOTU3 are unclassified bacterial and fungal species, respectively, and whether they are involved in SCN suppression needs further investigation. Additionally, we found that *N. punctiforme* inoculants significantly reduced the Shannon index of fungal community but had minimal effects on the bacterial diversities. The reduction of the Shannon index of fungal community may be attributed to the high abundance of some fungal taxa in the cyanobacteria inoculated samples, compared to the controls. Taken together, the changes in microbial communities and the increase in relative abundance of these potential parasitical microbial taxa in the soybean rhizosphere by *N. punctiforme* inoculants may contribute to the reduction of SCN population. But the suppressive functions of the specific bacterial and fungal species need further investigation.

Previous research showed that beneficial microorganisms, including cyanobacteria, generally elicit plant defense against biotic challenges (Abdelaziz et al., 2022; Molinari and Leonetti, 2019). The extracts of *Oscillatoria* sp., a cyanobacterium, regulated pathogenesis-related genes linked with plant defense and immunity against soybean root-rot nematode (Ghareeb et al., 2022). Belton et al. (2021) reported that *N. punctiforme* induced defense genes against programmed cell death in *Arabidopsis thaliana*. However, in this study, the expression levels of five soybean *PR* genes were examined by qPCR in the soybean roots of *N. punctiforme* and SCN treated, SCN only, and the controls without *N. punctiforme* and SCN inoculations. The results showed that *N. punctiforme* inoculants did not significantly upregulate or downregulate the five *PR* genes compared with SCN-treated only, suggesting *N. punctiforme*-enhanced soybean resistance to SCN may not be directly associated with the induction of soybean defense system. However, our current data does not exclude the possibility of indirect interaction with other soil microbes that might activate plant defenses.

**Figure 6:**
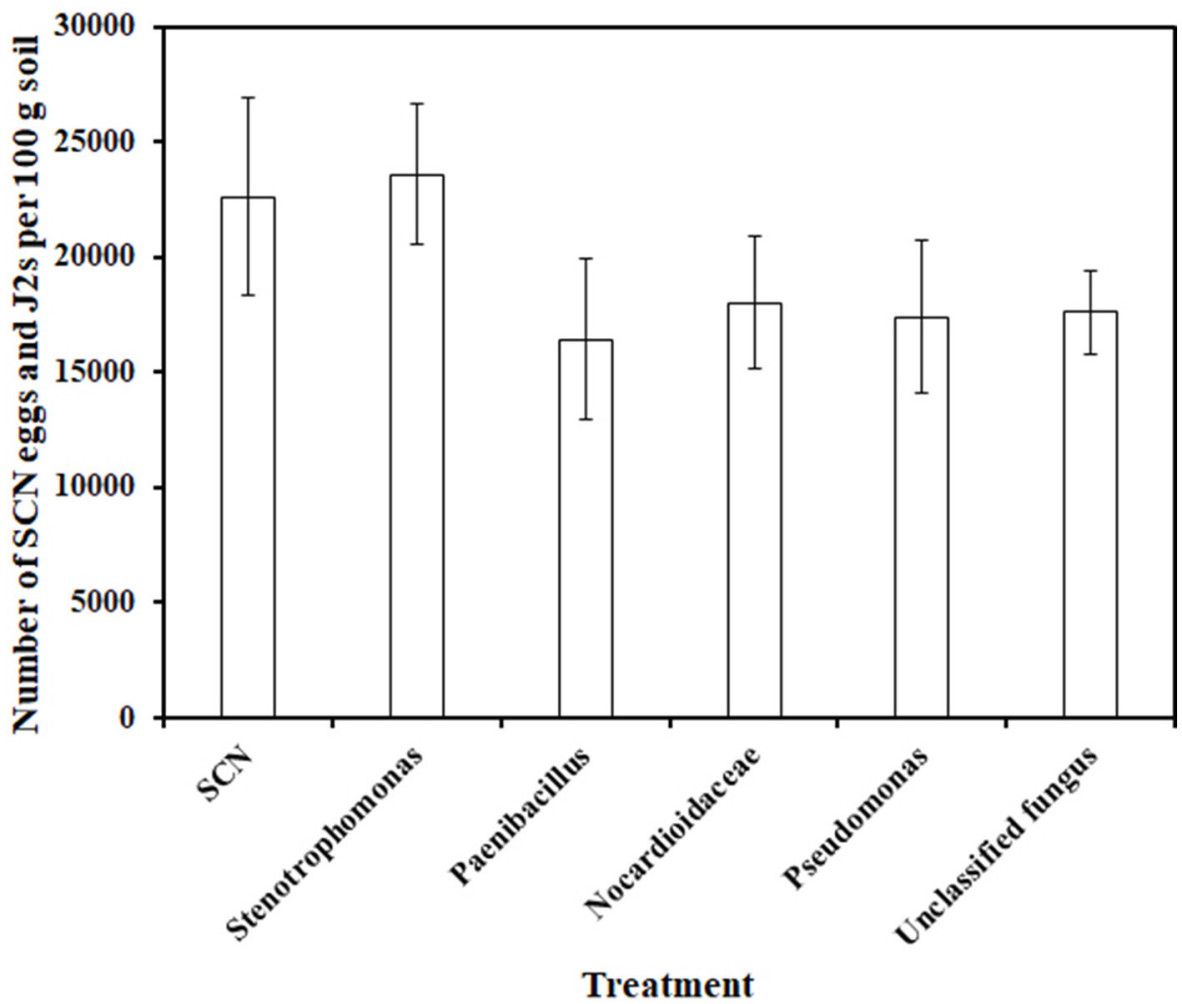
Effect of *N. punctiforme* co-existing microbial inoculants on soybean cyst nematode. SCN: SCN inoculation, Stenotrophomonas: SCN and *Stenotrophomonas maltophilia* inoculation, Paenibacillus: SCN and *Paenibacillus lactis* inoculation, Nocardioides: SCN and *Nocardioides zeae* inoculation, Pseudomonas: SCN and *Pseudomonas sp*. inoculation, Unclassified fungus: SCN and unclassified fungus inoculation (Kruskal-Wallis's test, *p* = 0.72, *n* = 4).

Many filamentous cyanobacteria possess a complex multilayer envelope around the cells, making it difficult to isolate single filaments that are free of other associated microbes (Fernandez-Valenzuela et al., 2021). Due to *N. punctiforme* generally being wrapped by extracellular polysaccharide sheaths (Sharma et al., 2009), obtaining axenic cultures of *N. punctiforme* is challenging. In this study, we did not successfully obtain axenic cultures of *N. punctiforme*, although several available methods were used, including repeated transfer of cells (Rippka et al., 1981), antibiotic, thermal, and sonication treatments (Han et al., 2014; Meixner et al., 2022), and treating akinetes with sodium hypochlorite (Šulčius et al., 2017; Vaz et al., 2014). To examine whether these co-existing microbes influence SCN population, one fungal and four bacterial strains were isolated from the *N. punctiforme* cultures using tryptic soy agar and potato dextrose agar media, respectively. Those microbial strains were identified as *Stenotrophomonas maltophilia, Paenibacillus lactis, Nocardioides zeae, Pseudomonas* sp., and unclassified fungus, respectively, using 16S rRNA and ITS primers (Yin et al., 2024). The cultures of single bacterial and fungal strain inoculations did not significantly change the total number of SCN eggs and J2s associated with susceptible “Williams 82” compared with the SCN inoculation only (Figure 6), indicating the SCN suppression activity of *N. punctiforme*. However, we cannot exclude the potential role of consortium of *N. punctiforme* and coexisting microbes in SCN infection.

The establishment and persistence of the introduced microbes in soil are important for functioning effectively. The microbial data in this study showed that ZOTU42 was more abundant in *N. punctiforme*-treated samples than in the controls (SCN inoculation only). The v4 hypervariable region of 16S rRNA sequence of ZOTU42 shows 100% query cover and identity to the sequence of *N. punctiforme* suggesting that the presence of inoculated cyanobacterial strain in the soil 5 weeks post inoculation. However, *N. punctiforme* grows relatively slowly (about 4 weeks to reach OD_720_ = 0.5) in the test condition that may limit the application of *N. punctiforme* on a large scale in fields.

## Data Availability

The datasets presented in this study can be found in online repositories. The names of the repository/repositories and accession number(s) can be found below: https://www.ncbi.nlm.nih.gov/, PQ740210 and project PRJNA1196987.

## Supplementary material

Supplementary Figure 1 Cyanobacterium isolation and purification. **(A)** *Nostoc punctiforme* growth in BG11 liquid media, **(B)** The filamentous and heterocytes of *N. punctiforme* under 100 x microscopy. Red arrows indicate heterocytes.

Supplementary Figure 2 Effect of *Nostoc punctiforme* inoculants on soybean cyst nematode. **(A)** Independent experiment 1, **(B)** Independent experiment 2. CK: control without SCN (HG type 7) and *N. punctiforme* inoculation, SCN: SCN inoculation, Low Conc: SCN and low concertation *N. punctiforme* inoculation, High Conc: SCN and high concertation *N. punctiforme* inoculation. Different letters indicate a statistically significant difference between treatments within an experiment as determined by Kruskal-Wallis's test (*p* ≤ 0.05, *n* = 4).

Supplementary Figure 3 Effect of *Nostoc punctiforme* inoculants on soybean growth. **(A)** soybean shoot length, **(B)** soybean shoot fresh weight. CK: control without SCN and *N. punctiforme* inoculations, SCN: SCN inoculation, Low Conc: SCN and low concertation *N. punctiforme* inoculations, High Conc: SCN and high concertation *N. punctiforme* inoculations. Different letters indicate a statistically significant difference between treatments within an experiment as determined by the Kruskal-Wallis's test (*p* ≤ 0.05, *n* = 4).

Supplementary Figure 4 Microbial taxonomic assignments at the phyla level and their percentage contribution (abundance) in the soybean rhizosphere. **(A)** bacterial phyla, **(B)** fungal phyla. SCN: SCN inoculation, Low Conc: SCN and low concertation *N. punctiforme* inoculations, High Conc: SCN and high concertation *N. punctiforme* inoculations.

## References

[B1] Abdelaziz A. M. Attia M. S. Salem M. S. Refaay D. A. Alhoqail W. A. Senousy H. H. (2022). Cyanobacteria-mediated immune responses in pepper plants against *Fusarium Wilt*. Plants 11:2049. 10.3390/plants11152049 35956527 PMC9370725

[B2] Allen T. W. Bradley C. A. Sisson A. J. Byamukama E. Chilvers M. I. Coker C. M.. (2017). Soybean yield loss estimates due to diseases in the United States and Ontario, Canada, from 2010 to 2014. Plant Health Prog. 18, 19–27. 10.1094/PHP-RS-16-0066

[B3] Alvarez A. L. Weyers S. L. Gardner R. D. (2024). Cyanobacteria-based soil amendments in the soil-plant system: effects of inoculations on soil nutrient and microbial dynamics under spring wheat growth. Algal Res. 77:103326. 10.1016/j.algal.2023.103326

[B4] Alves L. C. De Souza J. A. M. de Mello Varani A. de Macedo Lemos E. G. (2014). “The Family Rhizobiaceae,” in The Prokaryotes eds. Rosenberg E. DeLong E. F. Lory S.. (Berlin, Heidelberg: Springer), 419–437. 10.1007/978-3-642-30197-1_297

[B5] Arjoune Y. Sugunaraj N. Peri S. Nair S. V. Skurdal A. Ranganathan P.. (2022). Soybean cyst nematode detection and management: a review. Plant Methods 18:110. 10.1186/s13007-022-00933-8 36071455 PMC9450454

[B6] Asimakis E. Shehata A. A. Eisenreich W. Acheuk F. Lasram S. Basiouni S.. (2022). Algae and their metabolites as potential bio-pesticides. Microorganisms 10:307. 10.3390/microorganisms10020307 35208762 PMC8877611

[B7] Bandara A. Y. Weerasooriya D. K. Bradley C. A. Allen T. W. Esker P. D. (2020). Dissecting the economic impact of soybean diseases in the United States over two decades. PLoS One 15:e0231141. 10.1371/journal.pone.0231141 32240251 PMC7117771

[B8] Belton S. McCabe P. F. Ng C. K. (2021). The cyanobacterium, *Nostoc punctiforme* can protect against programmed cell death and induce defence genes in *Arabidopsis thaliana*. J. Plant Interact. 16, 64–74. 10.1080/17429145.2021.1891306

[B9] Bent A. F. (2022). Exploring soybean resistance to soybean cyst nematode. Annu. Rev. Phytopathol. 60, 379–409. 10.1146/annurev-phyto-020620-120823 35587510

[B10] Biondi N. Piccardi R. Margheri M. C. Rodolfi L. Smith G. D. Tredici M. R. (2004). Evaluation of *Nostoc* strain ATCC 53789 as a potential source of natural pesticides. Appl. Environ. Microbiol. 70, 3313–3320. 10.1128/AEM.70.6.3313-3320.2004 15184126 PMC427721

[B11] Boutte C. Grubisic S. Balthasart P. Wilmotte A. (2006). Testing of primers for the study of cyanobacterial molecular diversity by DGGE. J.Microbiol. Methods 65, 542–550. 10.1016/j.mimet.2005.09.017 16290299

[B12] Caporaso J. G. Lauber C. L. Walters W. A. Berg-Lyons D. Lozupone C. A. Turnbaugh P. J. Noah Fierer N. Knight R. (2011). Global patterns of 16S rRNA diversity at a depth of millions of sequences per sample. Proc. Natl. Acad. Sci. U.S.A. 108, 4516–4522. 10.1073/pnas.1000080107 20534432 PMC3063599

[B13] Chaudhary V. Prasanna R. Nain L. Dubey S. Gupta V. Singh R.. (2012). Bioefficacy of novel cyanobacteria-amended formulations in suppressing damping off disease in tomato seedlings. World J. Microbiol. Biotechnol. 28, 3301–3310. 10.1007/s11274-012-1141-z 22869418

[B14] Chen L. Cai Y. Liu X. Guo C. Sun S. Wu C.. (2018). Soybean hairy roots produced *in vitro* by *Agrobacterium rhizogenes*-mediated transformation. Crop J. 6, 162–171. 10.1016/j.cj.2017.08.00625097075

[B15] Chen S. Liu S. (2007). Effects of tillage and crop sequence on parasitism of *Heterodera glycines* juveniles by *Hirsutella* spp. and on juvenile population density. Nematropica 37, 93–106. Available online at: https://journals.flvc.org/nematropica/article/view/64419

[B16] Choleva B. Bileva T. Tsvetkov J. (2007). Organo-biological means and methods for control of plant parasitic nematodes as alternative of agrochemicals. Ecol. Fut. 6, 43–49. Available online at: https://www.cabidigitallibrary.org/doi/pdf/10.5555/20103006862

[B17] Cole J.R. Wang Q. Fish J.A. Chai B. McGarrell D.M. Sun Y.. (2014). Ribosomal database project: data and tools for high throughput rRNA analysis. Nucleic Acids Res. 42(D1), D633-D642. 10.1093/nar/gkt1244 24288368 PMC3965039

[B18] Deng X. Zhang N. Shen Z. Zhu C. Liu H. Xu Z.. (2021). Soil microbiome manipulation triggers direct and possible indirect suppression against *Ralstonia solanacearum* and *Fusarium oxysporum*. NPJ Biofilms Microbiomes 7:33. 10.1038/s41522-021-00204-9 33846334 PMC8041757

[B19] Desaeger J. Wram C. Zasada I. (2020). New reduced-risk agricultural nematicides-rationale and review. J. Nematol. 52, 1–16. 10.21307/jofnem-2020-091 33829179 PMC8015323

[B20] Edgar R. C. (2010). Search and clustering orders of magnitude faster than BLAST. Bioinformatics 26, 2460–2461. 10.1093/bioinformatics/btq461 20709691

[B21] Edgar R. C. (2016). UNOISE2: Improved error-correction for Illumina 16S and ITS amplicon sequencing. BioRxiv. 10.1101/081257

[B22] Edgar R. C. (2018). Updating the 97% identity threshold for 16S ribosomal RNA OTUs. Bioinformatics 34, 2371–2375. 10.1093/bioinformatics/bty113 29506021

[B23] Eken C. Uysal G. Demir D. Çalişkan S. Sevindik E. Çaglayan K. (2023). Use of *Arthrobotrys* spp. in biocontrol of the root-knot nematode Meloidogyne incognita. Eur. J. Biol. Res. 13, 173–180. 10.5281/zenodo.10015641

[B24] Fernandez-Valenzuela S. Chávez-Ruvalcaba F. Beltran-Rocha J. C. San Claudio P. M. Reyna-Martínez R. (2021). Isolation and culturing axenic microalgae: Mini-review. Open Microbiol. J. 15, 111–119. 10.2174/1874285802115010111

[B25] Ferris M. J. Hirsch C. (1991). Method for isolation and purification of cyanobacteria. Appl. Environ. Microbiol. 57, 1448–1452. 10.1128/aem.57.5.1448-1452.1991 16348486 PMC182968

[B26] Ghareeb R. Y. Abdelsalam N. R. El Maghraby D. M. Ghozlan M. H. El-Argawy E. Abou-Shanab R. A. (2022). *Oscillatoria* sp. as a potent anti-phytopathogenic agent and plant immune stimulator against root-knot nematode of Soybean cv. Giza 111. Front. Plant Sci. 13:870518. 10.3389/fpls.2022.870518 35720553 PMC9199862

[B27] Gohl D. M. MacLean A. Hauge A. Becker A. Walek D. Beckman K. B. (2016b). An optimized protocol for high-throughput amplicon-based microbiome profiling. Protoc. Exch. 10.1038/protex.2016.03028735410

[B28] Gohl D. M. Vangay P. Garbe J. MacLean A. Hauge A. Becker A.. (2016a). Systematic improvement of amplicon marker gene methods for increased accuracy in microbiome studies. Nat. Biotech. 34, 942–949. 10.1038/nbt.3601 27454739

[B29] Haarith D. Kim D. G. Chen S. Bushley K. E. (2021). Growth chamber and greenhouse screening of promising *in vitro* fungal biological control candidates for the soybean cyst nematode (*Heterodera glycines*). Biol. Control. 160:104635. 10.1016/j.biocontrol.2021.104635

[B30] Hamouda R. A. El-Ansary M. S. M. (2017). Potential of plant-parasitic nematode control in banana plants by microalgae as a new approach towards resistance. Egypt. J. Biol. Pest Control 27, 165–172. Available online at: https://www.cabidigitallibrary.org/doi/pdf/10.5555/20173278343

[B31] Han P. P. Jia S. R. Sun Y. Tan Z. L. Zhong C. Dai Y. J.. (2014). Metabolomic approach to optimizing and evaluating antibiotic treatment in the axenic culture of cyanobacterium *Nostoc flagelliforme*. World J. Microbiol. Biotechnol. 30, 2407–2418. 10.1007/s11274-014-1666-4 24832956

[B32] Holajjer P. Kamra A. Gaur H. S. Dhar D. W. (2012). *In vitro* nematicidal activity of a terrestrial cyanobacterium, *Synechococcus nidulans*, towards plant-parasitic nematodes. Nematology 14, 85–92. 10.1163/138855411X578879

[B33] Holajjer P. Kamra A. Gaur H. S. Manjunath M. (2013). Potential of cyanobacteria for biorational management of plant-parasitic nematodes: a review. Crop Protect. 53, 147–151. 10.1016/j.cropro.2013.07.005

[B34] Hunter M. C. Smith R. G. Schipanski M. E. Atwood L. W. Mortensen D. A. (2017). Agriculture in 2050: recalibrating targets for sustainable intensification. Bioscience 67, 386–391. 10.1093/biosci/bix010

[B35] Jose S. Malla M. A. Renuka N. Bux F. Kumari S. (2024). Cyanobacteria-green microalgae consortia enhance soil fertility and plant growth by shaping the native soil microbiome of *Capsicum annuum*. Rhizosphere 30:100892. 10.1016/j.rhisph.2024.100892

[B36] Joshi H. Shourie A. Singh A. (2020). “Cyanobacteria as a source of biofertilizers for sustainable agriculture,” in Advances in cyanobacterial biology (Cambridge, MA: Academic Press), (pp. 385–396). 10.1016/B978-0-12-819311-2.00025-5

[B37] Kim J. D. (2006). Screening of cyanobacteria (blue-green algae) from rice paddy soil for antifungal activity against plant pathogenic fungi. Mycobiology 34, 138–142. 10.4489/MYCO.2006.34.3.138 24039487 PMC3769562

[B38] Koenning S. R. Wrather J. A. (2010). Suppression of soybean yield potential in the continental United States by plant diseases from 2006 to 2009. Plant Health Prog. 11:5. 10.1094/PHP-2010-1122-01-RS

[B39] Kolde R. (2015). R pheatmap: Pretty Heatmaps. R package version 1.0.8. Available online at: http://CRAN.R-project.org/package=pheatmap (accessed December 11, 2015).

[B40] Kuraganti G. Edla S. Pallaval V. B. (2020). “Cyanobacteria as biofertilizers: current research, commercial aspects, and future challenges,” in Advances in Plant Microbiome and Sustainable Agriculture. Microorganisms for Sustainability eds. Yadav A. Rastegari A. Yadav N. Kour D. (Singapore: Springer). 10.1007/978-981-15-3204-7_11

[B41] Lee S. M. Ryu C. M. (2021). Algae as new kids in the beneficial plant microbiome. Front. Plant Sci. 12:599742. 10.3389/fpls.2021.599742 33613596 PMC7889962

[B42] Li H. Toh R. Wei Y. Wang Y. Hu J. An S.. (2022). Microbiomes across root compartments are shaped by inoculation with a fungal biological control agent. Appl. Soil Ecol. 170:104230. 10.1016/j.apsoil.2021.104230

[B43] Love M. I. Huber W. Anders S. (2014). Moderated estimation of fold change and dispersion for RNA-seq data with DESeq2. Genome Biol. 15, 1–21. 10.1186/s13059-014-0550-8 25516281 PMC4302049

[B44] Meixner K. Daffert C. Bauer L. Drosg B. Fritz I. (2022). PHB producing cyanobacteria found in the neighborhood-their isolation, purification and performance testing. Bioengineering 9:178. 10.3390/bioengineering9040178 35447738 PMC9030849

[B45] Molinari S. Leonetti P. (2019). Bio-control agents activate plant immune response and prime susceptible tomato against root-knot nematodes. PloS One 14:e0213230. 10.1371/journal.pone.0213230 31794550 PMC6890175

[B46] Mwaheb M. A. Hussain M. Tian J. Zhang X. Hamid M. I. El-Kassim N. A.. (2017). Synergetic suppression of soybean cyst nematodes by chitosan and *Hirsutella minnesotensis* via the assembly of the soybean rhizosphere microbial communities. Biol. Control 115, 85–94. 10.1016/j.biocontrol.2017.09.011

[B47] Nordbring-Hertz B. (2004). Morphogenesis in the nematode-trapping fungus *Arthrobotrys oligospora*-An extensive plasticity of infection structures. Mycologist 18, 125–133. 10.1017/S0269915X04003052

[B48] Nübel U. Garcia-Pichel F. Muyzer G. (1997). PCR primers to amplify 16S rRNA genes from cyanobacteria. Appl. Environ. Microbiol. 63, 3327–3332. 10.1128/aem.63.8.3327-3332.1997 9251225 PMC168636

[B49] Okanya P. W. Mohr K. I. Gerth K. Jansen R. Müller R. (2011). Marinoquinolines A- F, pyrroloquinolines from *Ohtaekwangia kribbensis* (Bacteroidetes). J. Nat. Prod. 74, 603–608. 10.1021/np100625a 21456549

[B50] Oksanen J. Blanchette F. G. Kindt R. Legendre P. McGlinn P. R. O'Hara R. B.. (2024). Vegan: Community ecology package. Available online at: https://cran.r-project.org/web/packages/vegan/index.html (accessed August 28, 2024).

[B51] Ou Y. Penton C.R. Geisen S. Shen Z. Sun Y. Lv N.. (2019). Deciphering underlying drivers of disease suppressiveness against pathogenic *Fusarium oxysporum*. Front. Microbiol. 10:2535. 10.3389/fmicb.2019.02535 31781059 PMC6861331

[B52] Parmar P. Kumar R. Neha Y. Srivatsan V. (2023). Microalgae as next generation plant growth additives: Functions, applications, challenges and circular bioeconomy based solutions. Front. Plant Sci. 14:1073546. 10.3389/fpls.2023.1073546 37063190 PMC10101342

[B53] Prasanna R. Chaudhary V. Gupta V. Babu S. Kumar A. Singh R.. (2013). Cyanobacteria mediated plant growth promotion and bioprotection against *Fusarium wilt* in tomato. Eur. J. Plant Pathol. 136, 337–353. 10.1007/s10658-013-0167-x

[B54] Priya H. Prasanna R. Ramakrishnan B. Bidyarani N. Babu S. Thapa S.. (2015). Influence of cyanobacterial inoculation on the culturable microbiome and growth of rice. Microbiol. Res. 171, 78–89. 10.1016/j.micres.2014.12.011 25644956

[B55] Qiu Y. Gu L. Tian S. Sidhu J. Gibbons J. Van Den Top T.. (2019). Developmentally regulated genome editing in terminally differentiated n2-fixing heterocysts of Anabaena cylindrica ATCC 29414. BioRxiv 629832. 10.1101/629832

[B56] Ramakrishnan B. Maddela N. R. Venkateswarlu K. Megharaj M. (2023). Potential of microalgae and cyanobacteria to improve soil health and agricultural productivity: a critical view. Environ. Sci. Adv. 2, 586–611. 10.1039/D2VA00158F

[B57] Righini H. Francioso O. Martel Quintana A. Roberti R. (2022). Cyanobacteria: a natural source for controlling agricultural plant diseases caused by fungi and oomycetes and improving plant growth. Horticulturae 8:58. 10.3390/horticulturae8010058

[B58] Rippka R. Waterbury J. B. Stanier R. Y. (1981). “Isolation and purification of cyanobacteria: some general principles,” in The prokaryotes. A handbook on habitats, isolation and identification of bacteria, eds. Starr M. P. Stolp H. Truper H. G. Balows A. Schlegel H. C. (New York: Spring-Verlag), 212–220. 10.1007/978-3-662-13187-9_8

[B59] Rodríguez-Caballero G. Caravaca F. Alguacil M. M. Fernández-López M. Fernández-González A. J. Roldán A. (2017). Striking alterations in the soil bacterial community structure and functioning of the biological N cycle induced by *Pennisetum setaceum* invasion in a semiarid environment. Soil Biol. Biochem. 109, 176–187. 10.1016/j.soilbio.2017.02.012

[B60] Saravanakumar K. Li Y. Yu C. Wang Q.Q. Wang M. Sun J.. (2017). Effect of *Trichoderma harzianum* on maize rhizosphere microbiome and biocontrol of Fusarium Stalk rot. Sci. Rep. 7:1771. 10.1038/s41598-017-01680-w 28496167 PMC5431858

[B61] Senkovs M. Nikolajeva V. Makarenkova G. Petrina Z. (2021). Influence of *Trichoderma asperellum* and *Bacillus subtilis* as biocontrol and plant growth promoting agents on soil microbiota. Ann. Microbiol. 71, 1–10. 10.1186/s13213-021-01647-3

[B62] Sharma M. Rani N. Kamra A. Kaushik A. Bala K. (2009). Growth, exopolymer production and metal bioremoval by *Nostoc punctiforme* in Na and Cr (VI) spiked medium. J. Environ. Res. Dev. 4:372. Available online at: https://www.academia.edu/11937063/GROWTH_EXOPOLYMER_PRODUCTION_AND_METAL_BIOREMOVAL_BY_Nostoc_punctiforme_IN_Na_AND_Cr_VI_SPIKED_MEDIUM

[B63] Shepherd A. M. (1970). “Extraction and estimation of *Heterodera*,” in Laboratory Methods for Work with Plant and Soil Nematodes. eds. Southey J. F. (Technical Bulletin 2. Ministry of Agriculture, Fisheries, and Food: London), 23–33.

[B64] Sithole N. Gupta S. Dube Z. Ogbe A. Van Staden J. (2023). Algae and cyanobacteria-based biostimulants in controlling plant-parasitic nematodes: a sustainable approach for crop protection. Phytoparasitica 51, 803–813. 10.1007/s12600-023-01094-7

[B65] Soliman M. S. El-Deriny M. M. Ibrahim D. S. S. Zakaria H. Ahmed Y. (2021). Suppression of root-knot nematode *Meloidogyne incognita* on tomato plants using the nematode trapping fungus *Arthrobotrys oligospora* Fresenius. J. Appl. Microbiol. 131, 2402–2415. 10.1111/jam.15101 33837626

[B66] SoyStats (2020). 2018 Soy Highlights. Available online at: http://soystats.com/2018-highlights

[B67] Šulčius S. Slavuckytė K. Januškaitė M. Paškauskas R. (2017). Establishment of axenic cultures from cyanobacterium *Aphanizomenon flos-aquae* akinetes by micromanipulation and chemical treatment. Algal Res. 23, 43–50. 10.1016/j.algal.2017.01.006

[B68] Sun X. Liao J. Lu J. Lin R. Zou M. Xie B.. (2024). Parasitism of *Hirsutella rhossiliensis* on different nematodes and its endophytism promoting plant growth and resistance against root-knot nematodes. J. Fungi 10:68. 10.3390/jof10010068 38248977 PMC10820206

[B69] Suresh A. Soundararajan S. Elavarasi S. Oscar F. L. Thajuddin N. (2019). Evaluation and characterization of the plant growth promoting potentials of two heterocystous cyanobacteria for improving food grains growth. Biocatal. Agric. Biotechnol. 17, 647–652. 10.1016/j.bcab.2019.01.002

[B70] Tedersoo L. Sánchez-Ramírez S. Koljalg U. Bahram M. Döring M. Schigel D.. (2018). High-level classification of the Fungi and a tool for evolutionary ecological analyses. Fungal Divers. 90, 135–159. 10.1007/s13225-018-0401-038816458

[B71] Thapa S. Prasanna R. Ramakrishnan B. Mahawar H. Bharti A. Kumar A.. (2021). Microbial inoculation elicited changes in phyllosphere microbial communities and host immunity suppress *Magnaporthe oryzae* in a susceptible rice cultivar. Physiol. Mol. Plant Pathol. 114:101625. 10.1016/j.pmpp.2021.101625

[B72] Tylka G. L. Gebhart G. D. Marett C. C. Mullaney M. P. Rasmussen J. (2022). 2022 Evaluation of Iowa soybean varieties resistant to soybean cyst nematode. Iowa State University, Extension and Outreach. Available online at: https://books.google.com/books?id=aE2mzwEACAAJ

[B73] Tylka G. L. Marett C. C. (2021). Known distribution of the soybean cyst nematode, *Heterodera glycines*, in the United States and Canada in 2020. Plant Health Prog. 22, 72–74. 10.1094/PHP-10-20-0094-BR

[B74] Vaz M.G.M.V. Bastos R.W. Milanez G.P. Moura M.N. Ferreira E.G. Perin C.. (2014). Use of sodium hypochlorite solutions to obtain axenic cultures of *Nostoc* strains (Cyanobacteria). Braz. J. Botany 37, 115–120. 10.1007/s40415-014-0055-4

[B75] Wang N. Tian S. Gu L. Xu L. Qiu Y. Van Den Top T.. (2018). Isolation of potential photosynthetic N 2-fixing microbes from topsoil of native grasslands in South Dakota. In Proceedings of the South Dakota Academy of Science (Vol. 97, p. 117). South Dakota State University, 272. Available omline at: https://openprairie.sdstate.edu/nrm_pubs/272

[B76] Wickham H. (2016). Ggplot2: Elegant graphics for data analysis. New York, NY: Springer-Verlag. 10.1007/978-3-319-24277-4_9

[B77] Winter S. M. J. Rajcan I. Shelp B. J. (2006). Soybean cyst nematode: challenges and opportunities. Can. J. Plant Sci. 86, 25–32. 10.4141/P05-072

[B78] Wram C. L. Hesse C. N. Zasada I. A. (2022). Transcriptional changes of biochemical pathways in *Meloidogyne incognita* in response to non-fumigant nematicides. Sci. Rep. 12:9875. 10.1038/s41598-022-14091-3 35701527 PMC9197979

[B79] Yin C. Casa Vargas J. M. Schlatter D. C. Hagerty C. H. Hulbert S. H. Paulitz T. C. (2021). Rhizosphere community selection reveals bacteria associated with reduced root disease. Microbiome 9, 1–18. 10.1186/s40168-020-00997-5 33836842 PMC8035742

[B80] Yin C. Larson M. Lahr N. Paulitz T. (2024). Wheat rhizosphere-derived bacteria protect soybean from soilborne diseases. Plant Dis. 10.1094/PDIS-08-23-1713-RE 38105448

[B81] Yu Z. Mo M. Zhang Y. Zhang K. Q. (2014). “Taxonomy of nematode-trapping fungi from *Orbiliaceae*, Ascomycota,” in Nematode Trapping Fungi. eds. Zhang K. Q. Hyde K. D. (Dordrecht: Springer), 41–210. 10.1007/978-94-017-8730-7_3

