# Supplementary figures and images for: Cyanobacterium Nostoc species mitigate soybean cyst nematode infection on soybean by shaping rhizosphere microbiota
Source: Front Microbiol. 2025 May 8;16:1544479. doi: 10.3389/fmicb.2025.1544479 (PMC12097279; doi:10.3389/fmicb.2025.1544479)

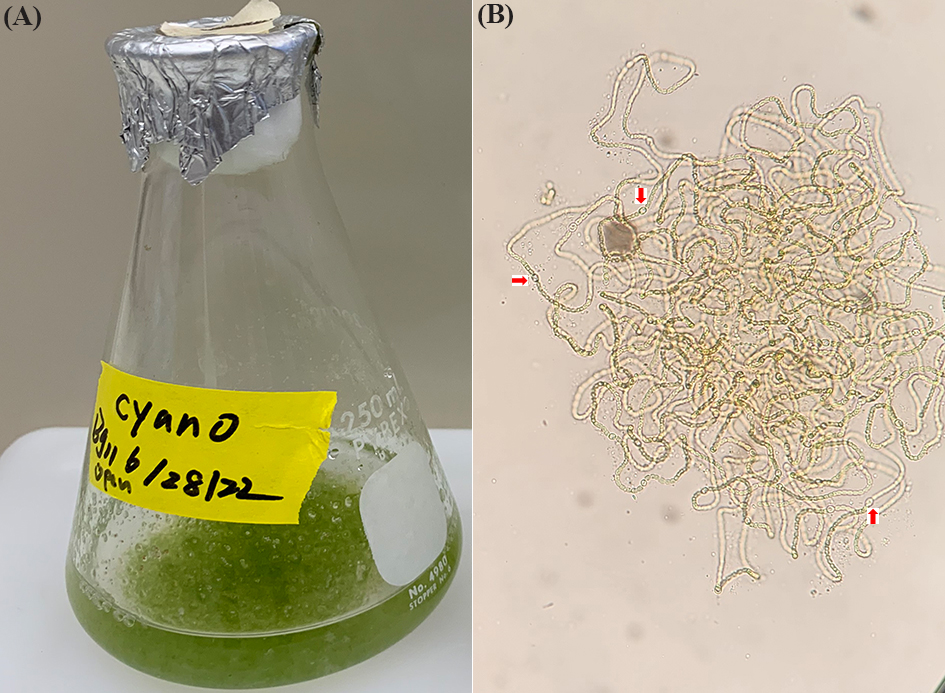

Supplement: Supplementary Figure 1 — Cyanobacterium isolation and purification. (A) Nostoc punctiforme growth in BG11 liquid media, (B) The filamentous and heterocytes of N. punctiforme under 100 x microscopy. Red arrows indicate heterocytes. [file Image_1.jpeg]

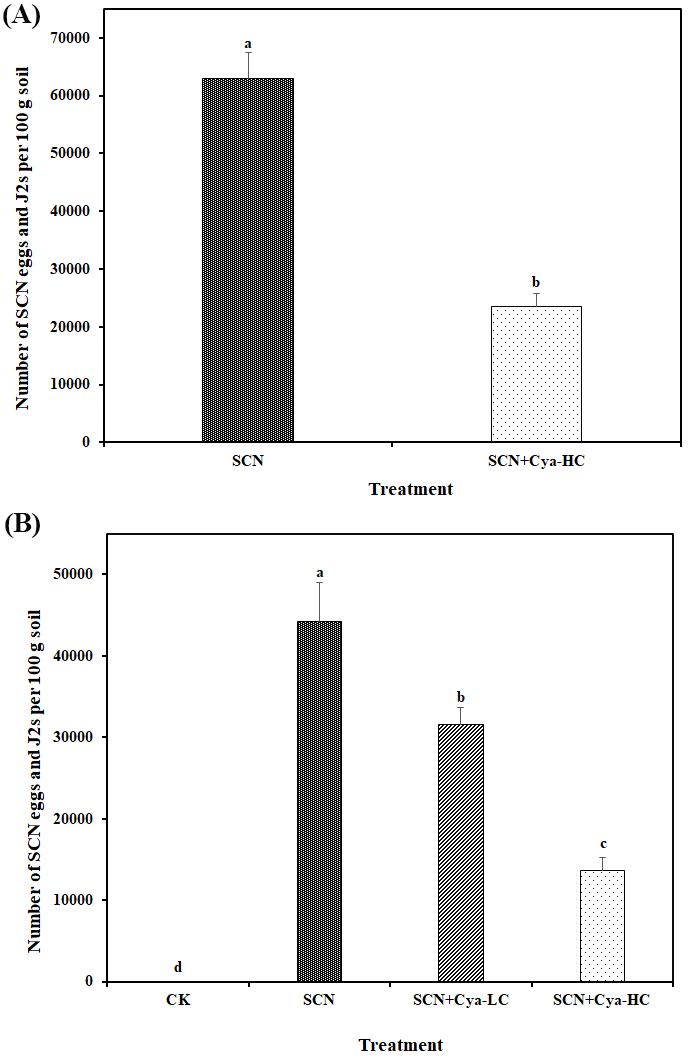

Supplement: Supplementary Figure 2 — Effect of Nostoc punctiforme inoculants on soybean cyst nematode. (A) Independent experiment 1, (B) Independent experiment 2. CK: control without SCN (HG type 7) and N. punctiforme inoculation, SCN: SCN inoculation, Low Conc: SCN and low concertation N. punctiforme inoculation, High Conc: SCN and high concertation N. punctiforme inoculation. Different letters indicate a statistically significant difference between treatments within an experiment as determined by Kruskal-Wallis's test (p ≤ 0.05, n = 4). [file Image_2.jpeg]

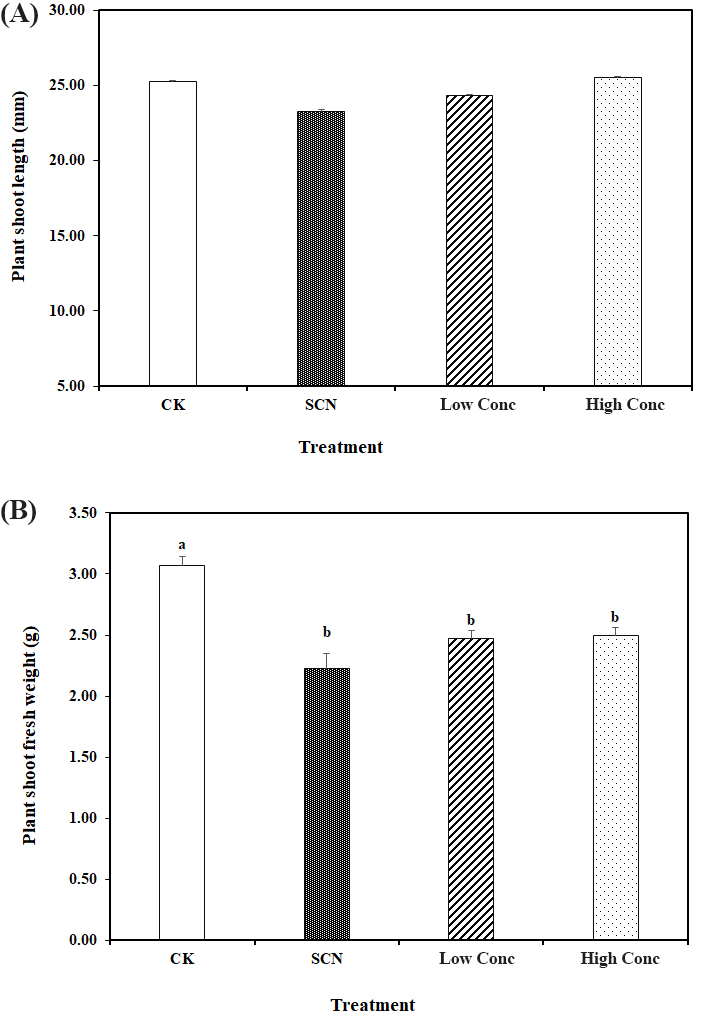

Supplement: Supplementary Figure 3 — Effect of Nostoc punctiforme inoculants on soybean growth. (A) soybean shoot length, (B) soybean shoot fresh weight. CK: control without SCN and N. punctiforme inoculations, SCN: SCN inoculation, Low Conc: SCN and low concertation N. punctiforme inoculations, High Conc: SCN and high concertation N. punctiforme inoculations. Different letters indicate a statistically significant difference between treatments within an experiment as determined by the Kruskal-Wallis's test (p ≤ 0.05, n = 4). [file Image_3.jpeg]

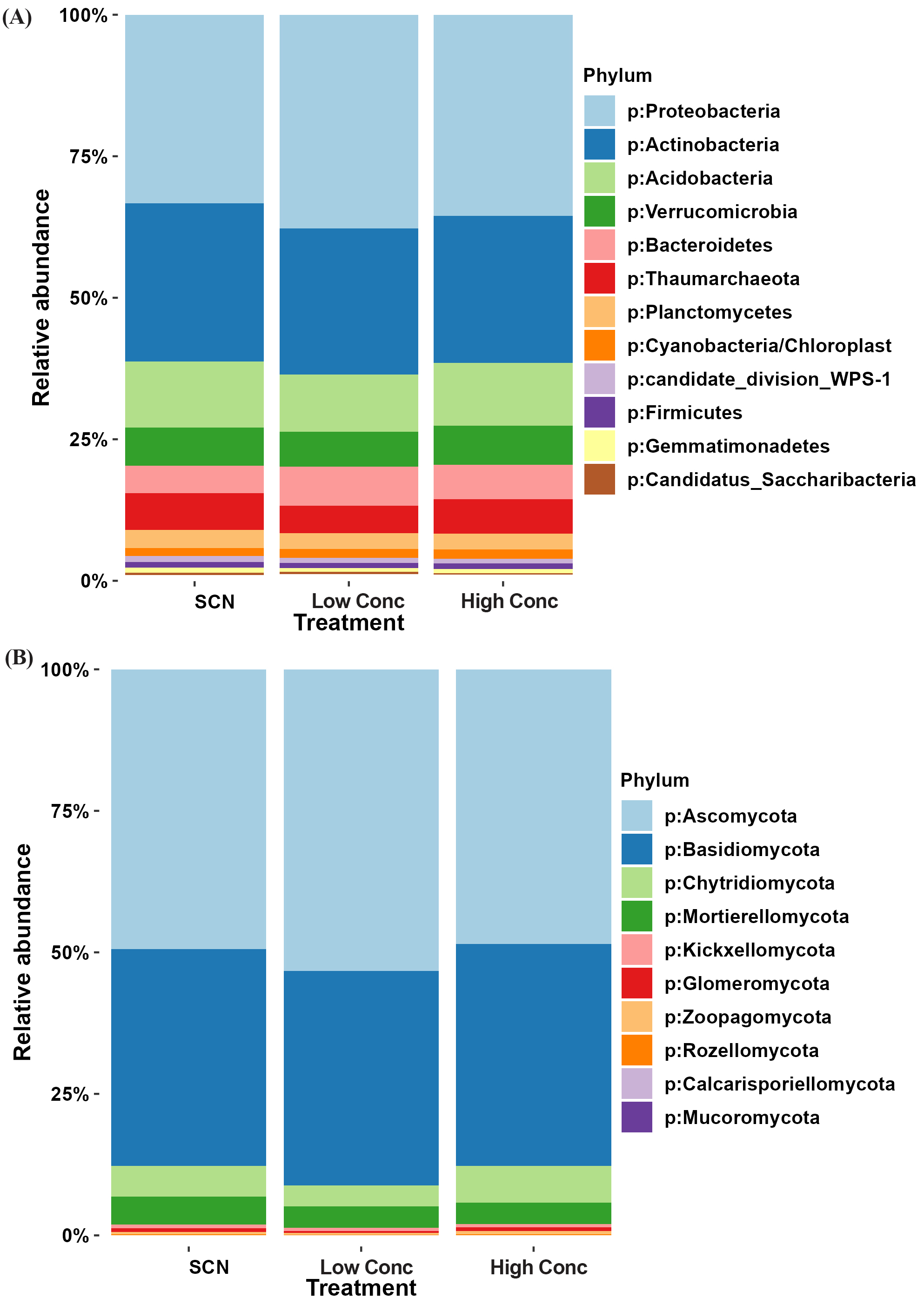

Supplement: Supplementary Figure 4 — Microbial taxonomic assignments at the phyla level and their percentage contribution (abundance) in the soybean rhizosphere. (A) bacterial phyla, (B) fungal phyla. SCN: SCN inoculation, Low Conc: SCN and low concertation N. punctiforme inoculations, High Conc: SCN and high concertation N. punctiforme inoculations. [file Image_4.jpeg]
